# Coastal barrier stratigraphy for Holocene high-resolution sea-level reconstruction

**DOI:** 10.1038/srep38726

**Published:** 2016-12-08

**Authors:** Susana Costas, Óscar Ferreira, Theocharis A. Plomaritis, Eduardo Leorri

**Affiliations:** 1 CIMA-University of Algarve, Faro, Portugal; 2 Department of Geological Sciences, East Carolina University, Greenville, NC, USA

## Abstract

The uncertainties surrounding present and future sea-level rise have revived the debate around sea-level changes through the deglaciation and mid- to late Holocene, from which arises a need for high-quality reconstructions of regional sea level. Here, we explore the stratigraphy of a sandy barrier to identify the best sea-level indicators and provide a new sea-level reconstruction for the central Portuguese coast over the past 6.5 ka. The selected indicators represent morphological features extracted from coastal barrier stratigraphy, beach berm and dune-beach contact. These features were mapped from high-resolution ground penetrating radar images of the subsurface and transformed into sea-level indicators through comparison with modern analogs and a chronology based on optically stimulated luminescence ages. Our reconstructions document a continuous but slow sea-level rise after 6.5 ka with an accumulated change in elevation of about 2 m. In the context of SW Europe, our results show good agreement with previous studies, including the Tagus isostatic model, with minor discrepancies that demand further improvement of regional models. This work reinforces the potential of barrier indicators to accurately reconstruct high-resolution mid- to late Holocene sea-level changes through simple approaches.

Future rates of global sea-level rise will likely increase during the 21st century and exceed the rate observed during 1971–2010 according to all Representative Concentration Pathway scenarios, due to increases in ocean temperature and loss of mass from glaciers and ice sheets^1^. However, as suggested by geological records and tide gauges, and more recently proven by satellite altimetry^2^, the resultant change is far from displaying a generalized trend but will rather have a strong regional pattern, with some places experiencing significant deviations of local and regional sea-level change from the global mean change^1^. Therefore, detailed impacts will vary spatially from region to region and cannot be easily assessed through broad-scale models, which poses a substantial challenge for the future.

Regional sea-level variability on timescales of years to decades is dominated by the contribution of non-uniform ocean thermal expansion^2^ and a combination of elasto-gravity effects on the distribution of melt water from the ice sheets^3^,^4^, wind forcing^5^,^6^, changes in the thermohaline circulation^7^, or vertical land movements, including glacio-isostatic adjustment^8^. All these complexities explain why available Glacio-Isostatic Adjustment models (GIA models) do not necessarily fit with field observations. On the one hand, because research advances in this area have not yet been able to fully resolve the histories of the ice-sheets (i.e. Fennoscandian, Laurentide and Antarctic Ice-Sheet) and the uncertainties on Earth structure^9^; on the other hand, because of the limited number of observations and the uncertainties associated with the estimates of age and elevation of sea-level index points^10^. Therefore, understanding the causes and patterns of regional variability is crucial, in particular for improving sea-level predictions based on climate models and for mitigating potential impacts of rapid sea-level rise in vulnerable coastal areas^11^. This understanding can only be achieved if high quality geological records are used to extend relative sea level far beyond the limits of instrumental record, thus contributing to a better understanding of the causes of sea-level change^11^,^12^, assess the effect of human activities on current sea-level trends^12^,^13^,^14^,^15^,^16^, and determine regional sea-level change variability at different time scales.

Current sources of post-glacial and Holocene relative sea-level index points (SLIPs), e.g. coastal wetland foraminifera, diatoms and pollen, coral reefs, sea-cave speleothems and archeological data, have reproduced sea-level histories over the last thousands of years for many regions worldwide^12^. Alternative proxies such as coastal barrier deposits have been also explored as potential indicators of sea-level change^17^,^18^,^19^,^20^,^21^,^22^, showing promising results, which could be further explored.

Sea-level changes along the Atlantic coast of the Iberian Peninsula have been analysed over the last three decades^23^,^24^,^25^,^26^,^27^,^28^,^29^. Nevertheless, sea-level changes over the mid- and late Holocene remain poorly understood, showing discrepancies in terms of the timing of the sea-level highstand and the magnitude of sea-level changes. Investigations developed along the southern coast of the Iberian Peninsula based on estuarine sedimentation^30^,^31^,^32^ document significantly different sea-level rise trends for the mid- to late Holocene (i.e. sea-level rise rates 1.2–2.6 mm/yr) from those obtained at the northern and central coast (i.e. 0.3–0.7 mm/yr) that include basal peats^27^,^29^. Reconstructions from the southern coast based on indicators located outside estuarine systems^33^,^34^, suggest there are significant local differences, which are difficult to explain by isostasy alone^29^. These differences are not trivial in the context of the expected impact on Holocene shoreline evolution, and question the reliability of previously used indexes and therefore of some of the proposed sea-level curves.

This research aims to (i) provide a rigorous (and innovative) methodology for enhancing the worldwide capability of detailed analysis of coastal morphology and coastal evolution integrated with dating of reconstructed sea-level changes, and (ii) resolve uncertainty in mid- to late Holocene relative sea-level changes at southern Iberia by generating new detailed and high-quality sea-level index points (SLIPs). For that, we will further explore the potential of coastal barriers in preserving signals of relative sea-level change and serving as paleoclimate proxies. The reconstructed sea-level record will be inferred from the position of both former beach berms and beach-dune interfaces related to the growth (elongation and progradation) of a coastal spit, Troia Peninsula (Fig. 1), dated by optically stimulated luminescence (OSL), and covering the time period of interest for this work, viz. the mid- to late Holocene. The results will be compared with alternative sea-level reconstructions to further test the capability of the proposed approach. Additionally, the results are expected to contribute to the discussion regarding the relative importance of natural and anthropogenic forcing on current sea-level rise by defining the natural or inherited footprint observed through the mid- to late Holocene, and of potential future geomorphic changes that may occur in response to continued sea-level rise.

## Sea-level indicators and coastal barriers

Coastal barriers are shaped by the constant effect of winds, waves, tides and related currents. However, the elevation of wind- and wave-generated deposits depends on the relative mean sea level. Coastal barriers may evolve vertically in pace with sea-level rise if accommodation space and sediment availability can compensate the changes promoted by sea-level oscillations. This relationship supports the suitability of coastal barriers as possible indicators of sea-level and climate changes. Hillaire-Marcel and Fairbridge^35^ demonstrated the reliability of beach-ridge plains as indicators of glacio-isostatic uplift and climate variability. While their ability as indicators of glacio-isostatic uplift is related to the average elevation of the explored ridges through time and space, their value as climate variability indicators is related to superimposed second order oscillations on ridge elevation. The latter was associated by the authors with inter-decadal climate variability, which is in turn responsible for changes in storminess and thus on the maximum runup elevation that ultimately determines beach ridge elevation. However, the actual elevation of a beach ridge may also include the effect of winds, which may delete part of the wave-induced berm or increase the elevation of the resulting ridge by aeolian accumulation^36^. To reduce the variability associated with beach ridges, different works have suggested and used the elevation of swales (i.e. low valleys formed between two wave-built berm ridges or foredune beach ridges^37^), whose modern analogs show less vertical variability^20^,^38^. However, the latter does not account for changes in the progradation rate that may in turn increase the elevation of the swales^22^. To minimize the impact of these limitations, subsurface sediment facies boundaries are preferred as sea-level indicators^36^. Among the more commonly used are the boundaries between the aeolian and beach sediments, the foreshore and shoreface^20^, and the upper and lower shoreface^39^.

The limit between the dune and the beach has been proved to successfully mark sea-level changes^18^,^19^,^22^,^40^,^41^. Preferably, the identification of this limit should be done with support of GPR data to resolve the internal barrier stratigraphy and spatially map the transition between dune and beach sediments^18^,^41^. Examples in the literature describe this transition as a remarkably smooth plane related to a grain-size shift from sand and gravel beach sediments to dune sand^18^, or as onlap terminations related to the welding of berms onto the beach^22^,^40^,^41^. The use of the dune-beach contact as sea-level indicator showed a very good agreement with more traditional methods^18^,^40^,^41^. Still, the identification of these indicators may present some limitations due to the inherent ambiguity in determining the limit between the dune and the beach because both settings may not present contrasting grain sizes^37^, and the indicators may have large vertical uncertainties related to the variability of the relative water level, particularly during storms^42^.

The interface between the foreshore and the shoreface observed in GPR images has also been used and dated to infer sea-level trends^20^,^21^,^43^,^44^,^45^. Some of these studies have focused on proving the reliability of this marker as a proxy of sea-level position by comparing recorded morphologies (morphology-slope of the reflections) with the present-day morphology of the foreshore and shoreface^43^,^46^. Though less popular, the limit between the upper and the lower shoreface can also be useful because of the facies change^47^. A major limitation to these two indicators, particularly at those regions with relatively high tidal ranges, is the depth at which these boundaries appear within the stratigraphic record, which in many cases cannot be reached by using GPR. In addition, these indicators are not always represented and thus recorded by a morphological feature; e.g. some beaches have low-tide terraces featuring the foreshore limit, but this is not the case in many beaches where tides are less important for the morphology than waves^48^.

## Results

### Modern analogs

The first morphological feature assessed within the present-day beach was the elevation of the contact between the dune and the beach (Fig. 2), which was defined as the seaward limit of the vegetation of incipient dunes and mapped alongshore using the aerial photographs contemporary with the LiDAR dataset used to estimate the elevation of the mapped points. The measured elevation of this transitional element was 4.59 ± 0.30 m above mean sea level (MSL). Assessing the elevation of this limit documents some problems as dune scarps were relatively frequent alongshore. In addition, the presence of a scarp at the frontal foredune was found to affect the elevation of the incipient dune. In general, sections characterized by partially eroded frontal dunes appear associated with lower dune-beach contact elevations. Conversely, accreting foredunes have been found associated with greater elevations and smoother contacts with the adjacent beach. A second assessment to estimate the elevation of this limit included the estimate of the elevation of the maximum runup measured *in situ* following a storm event. The latter documents an average elevation of 4.58 ± 0.86 m above MSL for the limit between the dune and the adjacent beach, suggesting a good agreement between both approaches. This feature is highly dependent on the morphological variability of the beach with larger values usually recorded at steeper foreshores and with greater incident wave heights and periods^49^.

The assessed second morphological feature was the elevation of the present-day beach berm, which is defined by an inflexion in the beach profile that represents the upper limit of the beach face whose slopes ranged between 0.06 and 0.1. The elevation of the berm was extracted from the LiDAR dataset and surveyed beach profiles, and it was estimated at 3.78 ± 0.31 m above MSL. The results from theoretical non-storm runup document modal values between 1.5 and 2 m (i.e. elevations between 3.5 and 4 m above MSL, during spring tides) in agreement with the berm elevation and slightly lower than the maximum runup measured *in situ* following extreme wave conditions (Fig. 2), which are in turn less frequent and more likely associated with erosive conditions.

### Sea-level indicators

A total of 77 onlap terminations interpreted as berm indicators, and 116 downlap terminations, representing the contact between the dune and the beach (dune-beach marker or indicator), have been digitized and extracted from the analyzed GPR line (Fig. 3 and Supplementary Fig. S1). Onlap terminations or markers were transformed into SLIPs using the elevations observed for the modern berm while downlap terminations were transformed into SLIPs by subtracting the estimate for the elevation of the modern beach-dune contact. The latter was chosen as it presented less variability than that obtained from the mapping of the maximum runup.

Dune-beach indicators show a higher variability than berm indicators (Fig. 3), in agreement with the greater variability observed for the present-day analogs. In this regard, it is worth noticing that despite the fact that the berm elevation may vary significantly depending on seasonal climate variability and the tides, recorded imprints of this feature are relatively stable, suggesting that only relatively high berms remain preserved within the barrier stratigraphy.

Indicators document a relatively constant rise in elevation across the barrier of approximately 2 m (Fig. 4). Relative sea-level rise (RSLR) estimates from both SLIPs indicate a steady rate of 0.31 ± 0.02 mm/yr (Fig. 4) for the last 6500 years. Despite this clear linear trend, small oscillations (<15 cm) can be observed in the curves. The latter have not been further explored as they are beyond the method resolution.

## Discussion

The stratigraphy of Troia Peninsula, a sandy spit located at the southwestern coast of the Iberian Peninsula, central Portugal, was explored here as a source of potential SLIPs to reconstruct relative sea-level trends over the past 6.5 ka. To do so, we have mapped two features found within the barrier stratigraphy marking the relative position of sea level from subsurface GPR images, topographically corrected with support of high-resolution GPS (RTK-GPS) and combined them with a chronological model based on the age of former shorelines obtained with the support of OSL and aerial photography. The first feature represents the contact between the upper beach and the dunes while the second morphological feature represents the upward limit of the beach face or the inflexion point from which the berm is built inland. In both cases, modern analogs were used to transform markers obtained from the stratigraphy into SLIPs as both features were associated to a specific present-day elevation range.

Indicators used to reconstruct RSLR require four attributes: location, age, elevation (both of the sampled indicator and the modern relationship with MSL), and tendency or indicative meaning of the indicator relative to the sea-level changes. Additionally, the chosen indicators must be able to accurately represent former sea levels^50^. The elevation of the contact between the dune and the beach responds linearly to sea level due to the limitation of dune plants to grow only above a particular elevation and distance from the shoreline^51^. Dune plant growth is hampered by the maximum penetration of moderate wave runup while storm waves may reduce incipient foredune life through erosion. The latter underlines the importance of wave climate for the vertical range of this indicator. However, as dune growth occurs over large time scales (years), the signal will represent an average of the wave climate shaping the coast rather than just the effect of rare events. In addition, it has been observed that the elevation of the dune contact may also vary depending on the morphology of the frontal dune; namely, on its erosive or accretionary character. The dune-beach contact at coastal sections with retreating frontal dunes was marked by lower elevations with scarps generated by storm erosion, which in turn provokes the lowering of the dune toe^52^. The inverse was observed at those sectors where the frontal dune is prograding. The longshore mapping of this transition captures not only a greater diversity of morphologies but also the response to wave height gradients as the wave energy increases to the south. Thus the modern analogs covered the full range of palaeoenvironments^50^.

The elevation of the berm depends on the tidal range and the magnitude of the incident waves. Indeed, higher berms form within the range of theoretical modal non-storm runup values as this feature can be described as a function of breaking wave height and period^53^. However, it is worth noticing as well that berm erosion is driven by large storm waves and in particular, associated infragravity waves^54^. Regarding tidal range, its effect is reflected in the vertical aggradation of the upper beach recorded within the barrier stratigraphy^55^, which supports the upper beach aggradation as a result of berm transport inland and upwards as tide range increases^56^. The latter suggests that high tide features are more likely preserved within the geological record if progradation is occurring.

The error of the modern analogs identified here is ca. ± 0.40 m for the dune-beach contact and ca. ± 0.30 m for the beach berm elevation, while errors associated with transfer functions from salt marshes, which quantify the vertical relationships between indicator species and tide level, range from ± 0.05 to ± 1.6 m^50^. Errors reported for high-resolution studies covering the last 3000 years average ± 0.16 m^57^. However, errors associated with late-glacial and Holocene reconstructions range on average between ± 0.7 and 1.6 m^29^,^58^,^59^, supporting the great potential of these SLIPs in terms of error range to develop high-resolution sea-level curves.

In addition to the vertical errors, the age errors need to be considered as well. For late-glacial and Holocene sea-level curves based on C14 true ages lie somewhere in a time span of 100 and over 650 years^29^. High-resolution Common Era relative sea-level reconstructions based on global databases provide smaller errors (average of ca. ± 86 years^57^). However, when based on organic carbon, and since the effect of refractory carbon has not been fully addressed, the chronology can be potentially impacted^60^. The chronology presented here is derived from Costas *et al*.^55^ and has been extended to the present-day by including the shoreline position at 1958 and 2010 extracted from aerial photography^61^. Although, we acknowledge the limitations derived from the reduced number of OSL samples and the existing gaps centered around 4.9 ka and 2 ka, we are convinced that this chronology should not limit the analysis of the overall trend of the obtained sea-level curve over the mid- to late Holocene. However, further dating is needed to pinpoint detected low-amplitude sea-level changes from the GPR record.

Our reconstructions suggest a continuous but slow sea-level rise (ca. 0.31 ± 0.02 mm/yr) for the explored time interval with an accumulated rise of around 2 m for both proxies. Superimposed to this linear trend, small (<15 cm) oscillations have been observed. While these oscillations might reflect some hydrological changes, the limitation imposed by our chronology precludes further inferences as aforementioned. The greater sea-level rise rates observed within the earlier section of the sea-level curve (Fig. 4) could be related to the end of the melting of major ice-sheets while the acceleration observed during the last 70 years is consistent with historical data^57^.

Trends of sea-level rise from intermediate-field sites in Europe are characterized by glacio-isostatic changes and subsidence in response to melting of the British and Fennoscandian ice sheets since the last glacial maximum^62^. Despite its relatively small size, the British ice sheet created large variations in relative sea-level trends from north to south that have not been yet resolved by current models^63^. Recent reconstructions from Western Brittany suggest a sea-level rise of ca. 1 mm/yr after the inflection at ca. 6 ka^63^,^64^ while reconstructions from the western Netherlands document greater rates (8 m of sea-level rise after 7 ka) without a clear inflection point but a continuous attenuation rate. Goslin *et al*.^65^ suggested the slowdown of sea-level rise at ca. 7 ka in general agreement with reconstructions from North Spain^29^ and North Portugal^27^, reporting sea-level rise trends between 0.3 and 0.7 mm/yr for the last 7 ka. This reflects a clear north to south glacio-isostatic response that culminated with the 2 m sea-level rise over the last 6.5 ka observed at our study site, with a more complex hydro-isostatic response (generally perpendicular to shore but rather complex). Conversely, a reconstruction from southern France documents a rather different trend with faster sea-level rise rates (1.7 ± 0.1 mm/yr) between 7.5 and 4 ka followed by lower velocities (0.4 ± 0.1 mm/yr) after 4 ka^62^, which in turn suggests that different factors may control the Mediterranean region. Yet, none of the referred works have been able to address the differences between the model and the observations in the southern-most regions of the Iberian Peninsula and therefore assumed that tectonic uplift should also be a significant driver in this region. However, the complex isostatic response together with the potential tectonic uplift can only be resolved with additional local and regional sea-level curves.

Regionally, data from the Sado estuary reported a sea-level rise of 1.7 mm/yr based on data that covered between ca. 7.2 and 2.8 ka^28^. These results contradict the data from the Tagus that document a rapid sea-level rise from 12 to 7 ka BP and a negligible rise since then^66^,^67^. Similarly, data from the southernmost coast of Portugal (Quarteira) based on bivalves^34^ suggest sea-level attenuation at 7 ka BP, when it reached 2.5 m below present, and a definitive stabilization 5 ka ago after reaching its present position. Data from the Sado estuary^28^ do not seem to fit nearby reconstructions, nor provided SLIPs. Therefore, these data are not included in the following comparisons. Figure 5 summarizes our data, the SLIPs from the Tagus estuary^29^, Quarteira^34^ and the isostatic model for the Tagus estuary^29^. The isostatic model used to generate predictions of past sea-level changes has been described in detail elsewhere^68^,^69^ and has been previously tested in this coastal area by Leorri *et al*.^27^,^29^. The relatively short distance between the Tagus and Troia (less than 50 km) supports their comparison. Quarteira is included only as a reference. There is a clear and significant overlap with the Tagus data at ca. 6 ka, which supports our reconstruction. In addition, the isostatic model also overlaps our data fairly well with only two offsets (ca. 6 ka and ca. 3 ka) but well within the error ranges. In addition, the Quarteira SLIPs also overlap the Tagus data for the earlier period, our data and the isostatic model. This could support our previous claim that while the model accurately depicts the north to south trend, the hydro-isostatic component might need reevaluation. Alternatively, the fit of the Quarteira data could be artificial and the hydro-isostatic component may be compensated by tectonic uplift in the southern-most region. However, sea-level reconstructions from estuarine sediments also collected along the southern coast of the Iberian Peninsula (Gulf of Cadiz) found that the highstand phase, after 6.5 ka BP, was characterized by sea-level rise rates between 1.5 mm/yr^30^,^32^ and 2.6 mm/yr^31^. While those rates are significantly larger than expected, they do not support tectonic uplift as a major factor in the southern region and therefore Quarteira might also be considered in this regional comparison. Current data, therefore, support the use of proposed SLIPs to provide accurate sea-level curves. The only offset between our data and the Tagus data is located at ca. 3.6 and 2.5 ka BP. However, this could result from autocompaction, as indicated above, in the estuarine sediments or reflect tidal changes within the estuarine system^70^. While regionally, data from Quarteira, does not support that claim, this inflexion has been reported in several estuaries^70^,^71^,^72^ demanding further research.

Finally, we have compared our results with monthly averaged data from the Cascais tide gauge and found that the amplitude of the changes in our record are within the range of variability of the instrumental dataset. The latter supports the potential of our data to provide an accurate background value to the 20th-21st century RSLR. If we apply the Holocene trend derived from this study to understand the 20th century sea-level rise at Cascais, a new estimate can be provided. The rate for the 20^th^ century has been estimated to be 1.69 ± 0.17 mm/yr at Cascais^73^. Previous estimates based on the isostatic model alone suggested 0.77 mm/yr^27^. However, if we consider our estimates as background RSLR, the new 20^th^ century rate would be 1.38 mm/yr.

## Summary

Despite the importance of sea-level studies in the context of current climatic scenarios and 30 years of research in SW Europe, there is no consensus regarding the rate of sea-level rise over the last 7000 years. In order to improve our understanding of this key coastal driver, here we have explored alternative sea-level indicators extracted from the stratigraphy of coastal barriers dated by OSL in combination with aerial photographs, and developed a new sea-level curve for the last 6.5 ka. For that, we have used the morphological features representing the contact between the dune and the beach, and the beach berm from Troia Peninsula, central Portugal. In both cases, the elevation of the selected features was found to depend on the position of mean sea level. Modern analogs of the selected features were examined in order to estimate their range of elevation and to transform the indicators extracted or mapped from the GPR data into sea-level indicators. This approach provided accurate sea-level indicators (error of ±0.30–0.40 m), well within the accuracy of current sea-level proxies based on transfer functions (±0.05 to ±0.88 m^50^), significantly smaller than Holocene reconstructions (±0.7 and 1.6 m), and only surpassed by high-resolution studies limited to the last 3000 years or less (average ±0.16 m). It is also worth stating that the combination of ages obtained from OSL and from the mapping of aerial photographs accurately allowed extending the sea-level record to the present in order to improve our chronology. The results document a steady sea-level rise of 0.31 ± 0.02 mm/yr in very good agreement with previous fieldwork data and modeling data from the Tagus River (50 km to the north), despite two minor offsets (ca. 6 ka and 3 ka) with the isostatic model.

Previous works exploring indicators extracted from coastal barriers focused on coastal sections with (i) high progradation rates, allowing the preservation of a relatively simple barrier stratigraphy mostly controlled by accretionary features, or (ii) high contrast between dune and beach sediment grain size. Here, we prove that coastal barrier indicators can also be extracted from barriers with complex stratigraphies resulting from low progradation rates, and without a clear contrast between dune and beach sediments, if high-resolution subsurface images are available. Additionally, our work shows that using two indicators extracted from the same dataset, but representing different depositional environments, improves the robustness of the extracted indicators.

## Methodology

**Figure 1:**
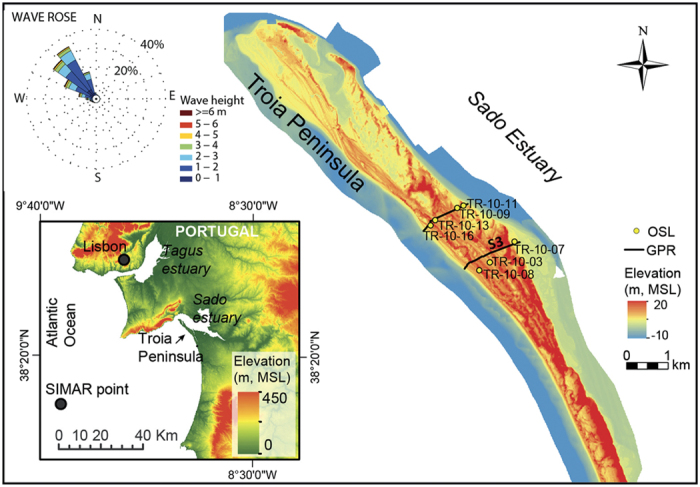
Location of Troia Peninsula, coastal barrier selected as case study, at the Portuguese coast (30 m resolution digital terrain model downloaded from http://www.arcgis.com/home/search.html?t=content&q=owner:ESRI-PT on 27 July 2010). GPR (Ground Penetration Radar) transects (solid black line) and OSL (optically stimulated luminescence) sample locations (yellow circles) are indicated over the digital terrain model elevation derived from the LiDAR dataset provided by the Direção-Geral do Território and the Agência Portuguesa do Ambiente. MSL – mean sea level. The wave rose was generated using hindcast wave data for the period between 1958 and 2015 (Simar dataset, code 1043054) provided by Puertos del Estado. The map was created using ArcGIS software version 10.3 (ESRI; http://www.esriportugal.pt/ArcGIS-for-Desktop).

Most of the coastal barriers along the Portuguese coast are very recent because of the retrograding character of the shoreline, which has induced the destruction of former barriers and the subsequent formation of new ones at landward positions through the mid- to late-Holocene^74^,^75^. Sheltered stretches of the coast, such as the littoral arc between Troia and Sines (Fig. 1), have high preservation potential of a long history of barrier elongation and progradation^55^. Costas *et al*.^55^ documented the growth history of Troia Peninsula based on OSL ages of beach and dune sediments. According to the authors, Troia is about 6.5 ka old and its formation was initiated after sea-level rise rate attenuation. Both, age (extending from the mid-Holocene to the present) and sheltering (to the dominant NW waves and storms), support Troia Peninsula as a suitable case study site for determining changes in relative sea level and testing the applicability of the proposed methodology to other coastal barriers.

Preferable sites for testing the application of SLIPs extracted from coastal barriers should include locations with high preservation potential in order to ensure that a large number of points or indicators can be obtained through time. Another key aspect to consider when scrutinising suitable sites is the magnitude of the runup, which is in turn mostly controlled by the exposure of the coast to the incoming waves and by the beach slope^49^. If possible, sites with reduced runup values are preferred to minimize vertical variability and reduce associated errors when compared with modern analogs. In this regard, sheltered areas are preferred sites as approaching waves have been significantly attenuated reducing the magnitude of runup values. Despite the general exposure of the Portuguese west coast to storms and waves with long periods, runup observed in the study area is reduced due to the sheltering effect of the cape located to the north. The shadowed zone created is receiving only a fraction of the offshore wave energy approaching from N-NW, which is in turn largely affected by wave refraction further reducing runup.

### Modern analogs

**Figure 2:**
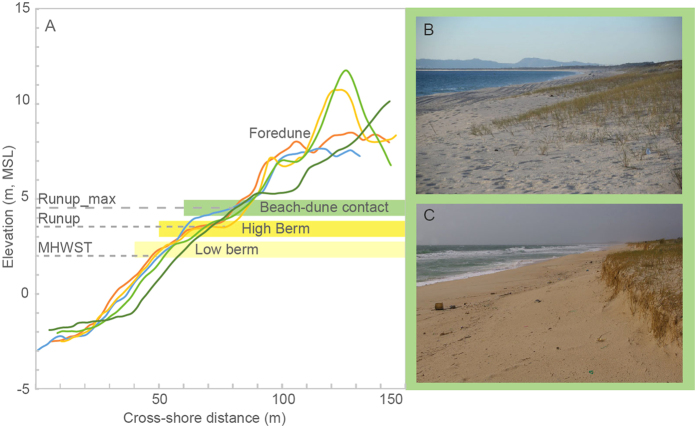
Examples of modern beach profiles extracted from the LiDAR dataset pointing out the location of the indicators used to track sea-level rise: the berm (high berm) and the contact between the dune and the beach (**A**). Note the presence of a second lower berm consequence of changes in wave climate and tidal cycles^55^. Pictures show the contact between the beach and the dune characterized by incipient vegetated dunes (**B**) and dune scarp (**C**). Mean high water during spring tides (MHWST), theoretical modal runup values calculated for non-storm conditions (Runup), and maximum runup values measured *in situ* following a major storm event (Runup_max).

**Figure 3:**
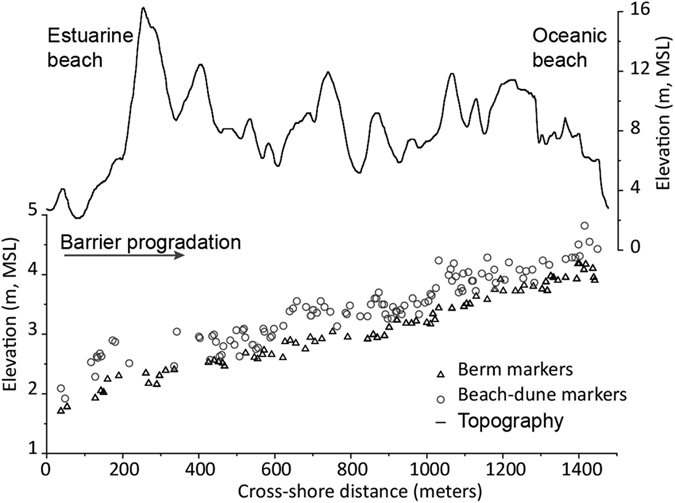
Plot showing the position and elevation of all subsurface markers extracted from GPR transect S3 (see Fig. 1), and the elevation of the barrier showing the size of the dune ridges.

**Figure 4:**
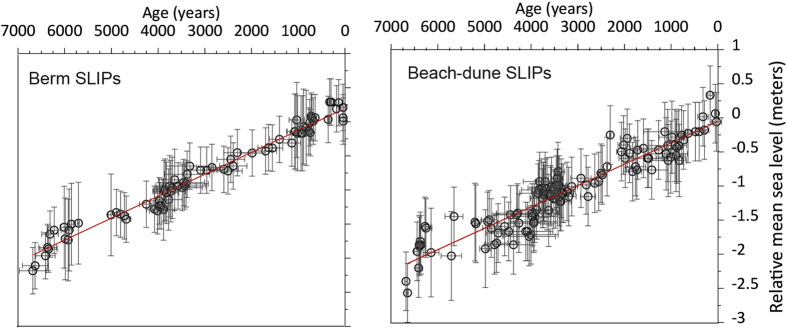
Representation of the SLIPs (berm and dune-beach indicators) extracted from the coastal barrier. The red line shows the linear adjustment of the data representing relative sea-level rise.

**Figure 5:**
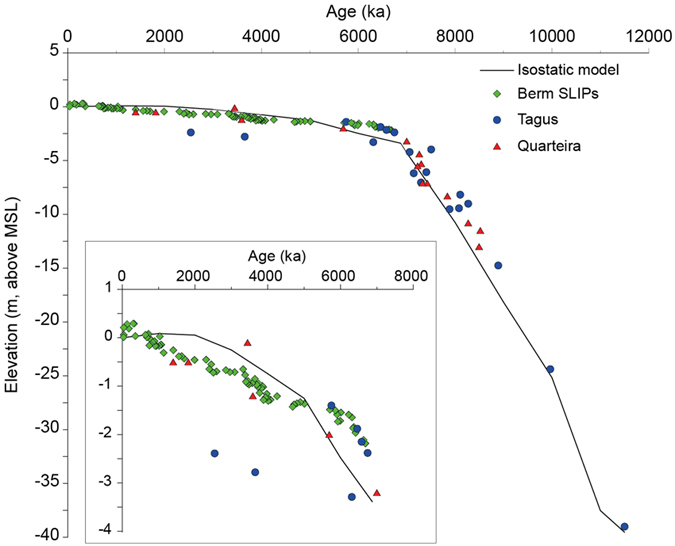
Plot comparing our data (berm indicators), the SLIPs from the Tagus estuary^66^, Quarteira^34^ and the isostatic model proposed by Leorri *et al*.^29^ for the Tagus estuary. The smaller panel shows the zoom into the time interval of focus of our study.

The elevation of the present beach-dune interface relative to MSL was estimated using two approaches: (i) the elevation of the limit between the beach and the dune using digital terrain models (e.g. LiDAR dataset), aerial photography, and ground-truthing, and (ii) the elevation of the maximum runup defined by the debris based on fieldwork surveys carried out following the impact of major storms (Fig. 2). The latter assumes that the dune is initiated in the lee of the beach debris or within the inland limit of the maximum penetration of the marine influence. In both cases, we have not restricted our observations to the area of the geological data (profiles on Fig. 1), but we have laterally extended our observations to capture modern morphological variability. For the first approach we mapped a total of 34 points along 2 km while for the second we mapped 6 points along 40 km in order to capture the morphological variability of selected features.

Present-day beach berm elevations were extracted from the LiDAR dataset (May/June 2011) and cross-shore beach profiles measured during the winter of 2010. In this case, we have measured three cross-shore beach profiles within an area of 1 km and extracted 18 points from the LiDAR dataset along 5 km of shoreline. Additionally, we have estimated the theoretical values of the non-storm or constructive runup using Holman^76^ equation and offshore hindcast wave data for the period between 1958 and 2015 (SIMAR dataset, code 1043054) provided by Puertos del Estado. Wave refraction and shoaling were calculated using linear wave theory. Wave directions greater than 310 degrees were not considered due to the presence of the headland to the north of the study site.

### Coastal barrier stratigraphy

GPR lines, running across the coastal barrier (Fig. 1), are here examined to map the best SLIPs and additional stratigraphic features to understand the evolution of the system in relation to sea-level and progradation rate changes. Subsurface images were acquired using an Ingegneria Dei Sistemi-Ground Penetrating Radar (IDS-GPR) system RIS MF Hi-Mod #1 equipped with a dual frequency antenna (200 and 600 MHz). Here, we have chosen the data provided by the lower frequency as it provided greater penetrations with relatively high vertical resolution (i.e. 0.14 m for a 230 MGz return centre frequency) within the upper limit of thin bedding^77^. Topographic corrections of the GPR data were obtained by using a RTK-DGPS, synchronized to the GPR during data acquisition, and applying a constant propagation velocity of 0.13 m/ns estimated using the interactive hyperbola-adaptation method. This correction does not account for depth wave attenuation that results in greater errors associated with the topographic correction of the transect across sections with higher dunes. The associated error was estimated considering the deformation of the water table observed within those sections where large dunes were surveyed.

**Figure 6:**
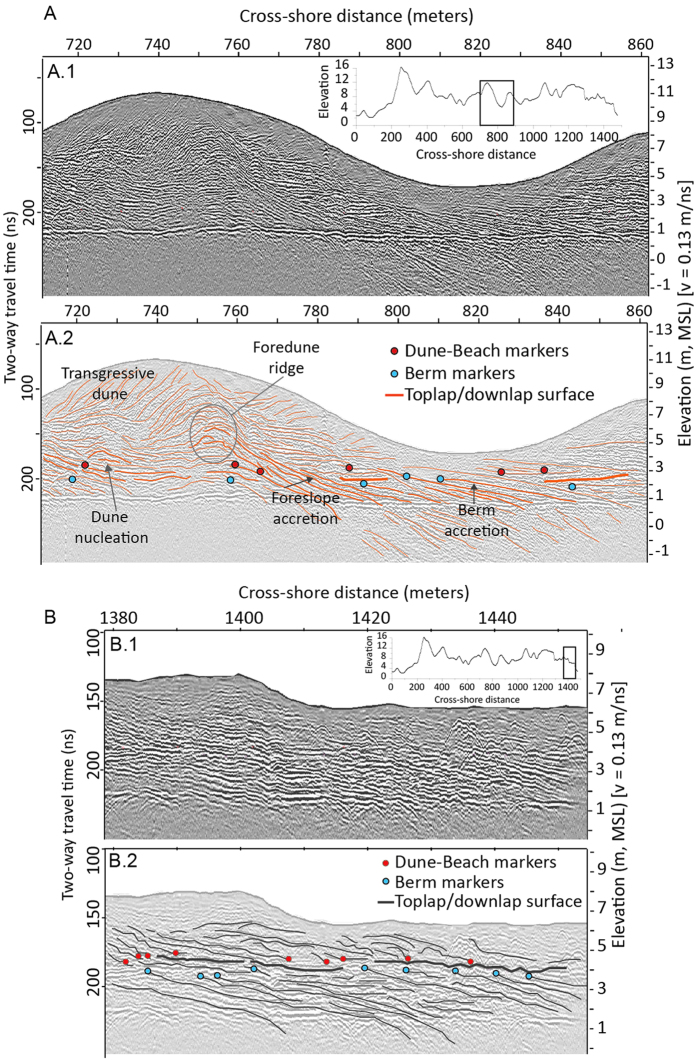
GPR sections including interpretation and identification of the selected indicators. Note the greater elevation of the indicators identified within the seaward section of the spit (B.2) relative to the ones found at the central section (A.2).

Radar facies assemblages within Troia stratigraphy have been described in detail in Costas *et al*.^55^. The authors identified two radar facies representing the upper beach (i.e. beach backshore and upper foreshore) and four radar facies representing the overlying aeolian deposits (i.e. vegetation nucleation, aeolian deflation, foreslope accretion, landward migration). The latter confirms the preservation of two potential indicators of sea-level position within the barrier stratigraphy. Namely, radar facies representing the elevation of the berm (berm indicators or markers) and characterized by onlap terminations of sigmoid-oblique reflections, and radar facies indicating the seaward progradation of the foredune and of the dune-beach contact (dune-beach indicator or marker) and represented by downlap terminations of tangential-oblique reflections (Fig. 6). Both indicators are examined and mapped across GPR line S3 (Fig. 1), which is the longest line and traverses the entire progradational history of the spit.

### Chronostratigraphy

**Figure 7:**
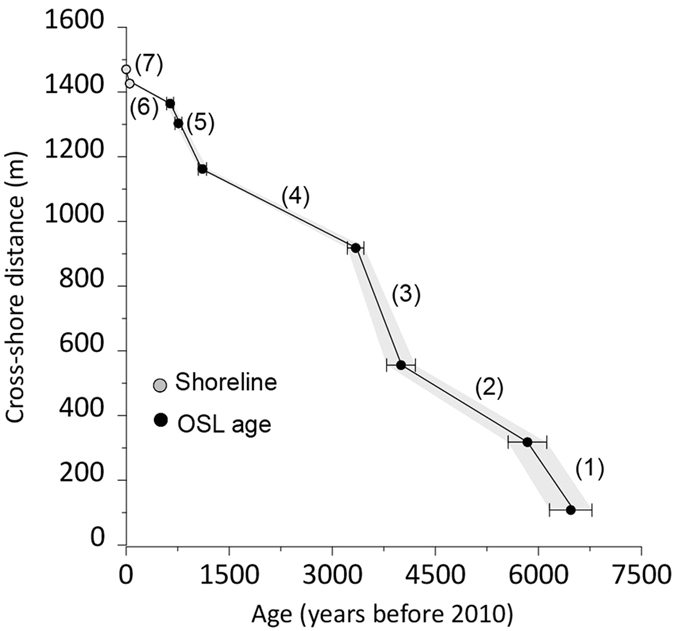
Chronostratigraphy of the profile running across the spit showing the linear adjustments (seven sections) based on the OSL ages of beach sediment samples^55^ and the shoreline position identified at 1958 and 2010^61^.

Seven OSL ages of beach sediments (Table 1), previously identified within the GPR lines (Fig. 1) and published in Costas *et al*.^55^, were used to estimate progradation rates and obtain an age model (Fig. 7). OSL dating analyses were made by ETN, C2TN, Instituto Superior Técnico. Field gamma spectrometry measurements were conducted *in situ* to obtain concentrations of K, Th, and U. In the laboratory, water content as a fraction of dry sample mass was measured as received, saturated, and following free drainage. Dose rates from alpha, beta and gamma radiation were calculated from elemental concentrations and corrected for time averaged water content^78^, which was constrained based on the measured values and the elevation of each sampling position relative to the water table. A more detailed description of the OSL dating analyses is provided in Costas *et al*.^55^.

The use of beach sediments ensures maximum age model reliability as it represents *in situ* sedimentation during barrier progradation. To improve our chronology, two more points have been added that represent the position of the shoreline in 1958 and 2010 extracted from aerial photography^61^.

Because the progradation history of the spit is not linear^55^, we have applied different progradation rates respecting substantial changes in the observed trends (Fig. 7). For that, we have defined the progradation rates of seven cross-shore sections using first order adjustments (Fig. 7). OSL age errors were considered to obtain the upper and lower ages for each SLIP and illustrate the horizontal error or variability of marker age estimates. Additionally, pits excavated for sample collection were used to calibrate GPR interpretations in terms of facies stratigraphy.

Once the markers are digitized and extracted from the GPR data (Fig. 6), they are assigned an age after applying the obtained age model defined by the cross-shore progradation rates (Fig. 7).

### Sea-level indicators

Extracted sea-level markers are transformed into SLIPs by subtracting the value of the modern analog (equation 1) to their elevation to represent the evolution of the mean sea level through time:


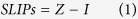


where Z is the elevation (i.e. in meters relative to MSL) of the digitized marker, and I is the indicative meaning of elevation of the present-day analog, also relative to MSL. For each index-point, the sum of the altitudinal error terms potentially introduced at each stage of the reconstruction can be calculated following equation 2 proposed by Horton *et al*.^79^:


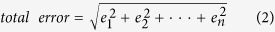


in this case, the total error is assumed to be the sum of the field levelling (±0.10 m); the extraction of the marker elevation from the radargram, which we assume that depends on the GPR resolution (±0.14 m) and on changes of the progradagation velocity of the electromagnetic waves within the barrier (±0.50 m) that maximizes at sections with large dune buildings; and the vertical range of variability of the modern analogs.

Once SLIPs are obtained, they were adjusted by applying a mid-point linear regression in order to estimate the mean RSLR rate through time and its standard deviation.

## Additional Information

**How to cite this article**: Costas, S. *et al*. Coastal barrier stratigraphy for Holocene high-resolution sea-level reconstruction. *Sci. Rep.* **6**, 38726; doi: 10.1038/srep38726 (2016).

**Publisher's note:** Springer Nature remains neutral with regard to jurisdictional claims in published maps and institutional affiliations.

## Supplementary Material

Supplementary Figure S1

## Figures and Tables

**Table 1:** OSL ages are represented in years before 2010 with 1σ uncertainties published in ref. 55.

Sample	ITNLUM#	Distance to present shoreline (m)	Elevation (m, MSL)	Burial Depth (m)	Burial average H2O (g/g)	Dose Rate (mGy/yr)	Absorbed Dose (Gy)	Age (yrs)
TR-10-03	665	544	6	0.90	0.06	1.13 ± 0.04	3.78 ± 0.06	3340 ± 120
TR-10-07	669	1440	3	1.00	0.09	0.95 ± 0.04	6.12 ± 0.15	6470 ± 300
TR-10-08	670	152	5	0.75	0.05	0.91 ± 0.03	0.70 ± 0.04	760 ± 50
TR-10-09	671	954	5	1.03	0.07	1.06 ± 0.04	4.26 ± 0.16	4000 ± 200
TR-10-11	673	1127	4	1.20	0.05	0.98 ± 0.03	5.74 ± 0.20	5840 ± 300
TR-10-13	675	362	5	1.70	0.07	1.03 ± 0.04	1.15 ± 0.05	1110 ± 60
TR-10-16	678	220	4	1.05	0.03	1.26 ± 0.04	0.81 ± 0.03	650 ± 30

Sample locations are shown in Fig. 1.
