# Supplementary material for: Coastal barrier stratigraphy for Holocene high-resolution sea-level reconstruction
Source: Sci Rep. 2016 Dec 8;6:38726. doi: 10.1038/srep38726 (PMC5144086; doi:10.1038/srep38726)
Supplement: Supplementary Figure S1 [file srep38726-s1.pdf]

Coastal barrier stratigraphy for Holocene high-resolution sea-level reconstruction

Susana Costas [1], Óscar Ferreira [1], Theocharis Plomaritis [1], Eduardo Leorri [2]

1. CIMA-University of Algarve, Faro, Portugal, 2. Department of Geological Sciences, East Carolina University, Greenville, NC

Supplementary figure

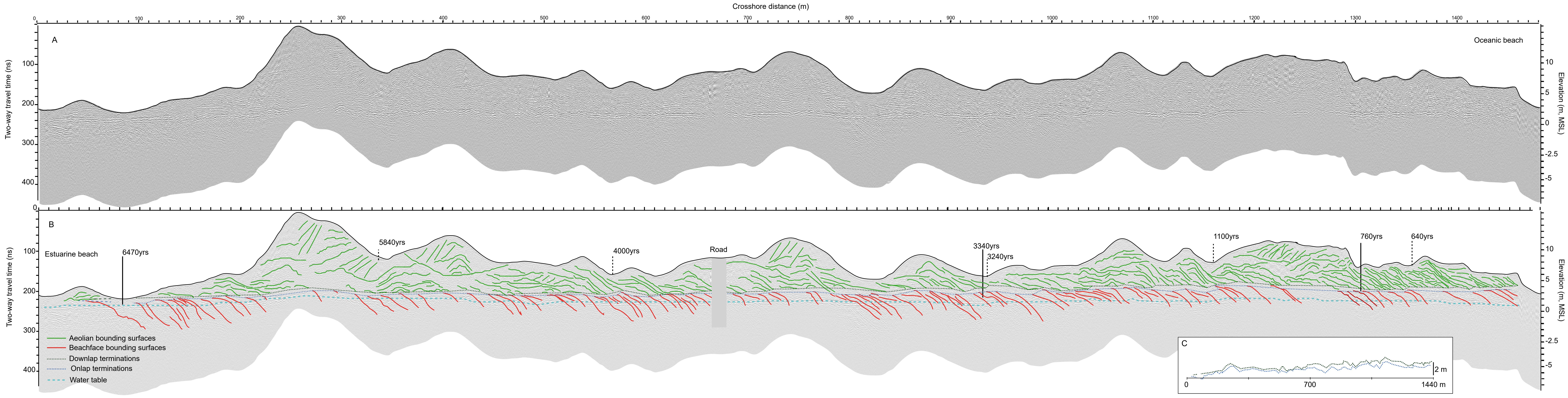

Supplentary Fig. S1 : GPR Transect including processed data (A) and interpretation (B) showing the location of the beach-dune, the upper part of the beachface, and OSL ages collected within GPR profile S3 (continuous line) and adjacent samples (discontinuous line). The elevation of both indicators is pointed out bytwo lines joining acrosshore the downlap and onlap terminations. The overall trend is summarized in C. See Figure 1 for OSL sample location.
